# Breastfeeding technique and associated factors among lactating mothers visiting Gondar town health facilities, Northwest Ethiopia: observational method

**DOI:** 10.1186/s13052-021-01158-6

**Published:** 2021-10-12

**Authors:** Boko Loka Safayi, Nega Tezera Assimamaw, Destaye Guadie Kassie

**Affiliations:** 1 Nursing Department, College of Health and Medical Sciences, Bule Hora University, Bule Hora, Ethiopia; 2 Department of Pediatrics and Child Health Nursing, College of Medicine and Health Sciences, School of Nursing, University of Gondar, P.O .Box:196, Gondar, Ethiopia

**Keywords:** Breastfeeding technique, Lactating mothers, Health facilities, Northwest Ethiopia

## Abstract

**Background:**

The breastfeeding technique is explained positioning, attachment, and suckling during breastfeeding. Ineffective breast feeding technique is one of the factors leading to premature discontinuation of breastfeeding and malnutrition. There is a limited study on the assessments of BFT and associated factors among lactating mothers in the study area. Therefore, the study aimed to assess breastfeeding technique and the associated factors among lactating mothers visiting Gondar town health facilities, Northwest Ethiopia.

**Methods:**

An institution-based cross-sectional study was conducted from February 20 to March 20, 2020. An observational checklist and structured interviewer-administered questionnaire were applied to collect the data. The data were entered into Epi-Data 4.6 version and transferred to analyzed using SPSS version 20. Bi-variable and multivariable logistic regressions were performed to identify the association. The strength of association was identified using odds ratio with a 95% confidence interval (), and a *p*-value of 0.05 was declare as statistically significant.

**Results:**

The proportion of effective BFT was 48% (95%, CI: 43.0–53.0%). The likelihood of applying proper BFT among age group of 18–20 years was 70% lower than (AOR = 0.3; 95% CI: 0.11–0.83) age group > 30 years. The odds of effective BFT in primipara mothers were 49% (AOR = 0.51; 95% CI: 0.32–0.82) lower compared to multiparous mothers. Likewise, the provability of effective BFT was 55% (AOR = 0.45; 95% CI: 0.29–0.70) times lower in women who had no counseling immediately after delivery compared to their counterparts. Moreover, effective breast feeding technique mothers who have breast problem was 78% lower than (AOR = 0.22; 95% CI: 0.07–0.68) mothers who have no breast problem. And mothers who had counselling of BFT during ANC follow up was 55% (AOR = 0.45; 95% CI: 0.29, 0.70) lower than mothers who had no counseling.

**Conclusion:**

Just under half of the women in the study area applied proper breast feeding technique Younger and primipara mothers poorly performed to effective BFT. But women having counseling during antenatal care follow-up and immediately after delivery and not having breast problems applied BFT effectively. Hence, special emphasis have to give for younger and primipara mothers. Besides, educate the mother for preventing breast problems and working on enhancing counseling at postnatal clinic.

## Background

Breastfeeding technique is explained as the mother’s and baby’s positioning, baby’s attachment to the breast, and suckling during breastfeeding, which are very important for effective breastfeeding [1–3]. Appropriate early childhood care and application of effective breastfeeding techniques during infancy are the key to optimum development, health, and survival [4–6]. The World Health Organization (WHO) has recommended to utilize the appropriate breastfeeding techniques that helps to promotes exclusive breastfeeding, due to this reason the baby can get the expected amount of energy and adequate nutrients. In addition, breast feeding is important for both the mothers and infants health with protecting against different acute and chronic disorders [7, 8].

Ineffective BFT is one of the main factors leading to non-EBF and premature discontinuation of breastfeeding [9]. Several evidences revealed that 58% of mothers with poor BFT cease breastfeeding earlier and interferes with EBF; which ultimately result in a serious of acute infectious diseases like diarrhea, acute respiratory infections, and chronic diseases; such as diabetes, a reduced mental ability, and perhaps an increased risk of epileptic disorders during childhood [6, 8, 10–13].

Other than the afore mentioned problems, poor BFT has profound impact on infant’s breast milk intake [9], which leads to the different forms of under nutrition, including macro and micronutrient deficiencies [14]. According to the United Nations, Children’s Emergency Fund (UNICEF), WHO, and World Bank joint child malnutrition estimated; inadequate food and breast milk intake are the primary and immediate causes of infant and young child under nutrition. More than half of the deaths of under-five children are due to malnutrition that the most public health problems in developing countries like Ethiopia [15]. Consistent adhering of WHO BFT helps the mother for preventing breast conditions like cracked, sore nipples and other risks [16].

Multiple research reports showed regarding to factors affect the effective BFT are young mothers, primiparity, illiterate mother, unemployed mother, breast diseases, previous breastfeeding experience, Antenatal Care (ANC) and Postnatal Care (PNC) counseling. Moreover infant factors that affect the Effective BFT includes gestational age, the current age of the infant, and bottle feeding [2, 10, 14, 17, 18].

The Baby-Friendly Hospital Initiative and WHO designed the counseling, teaching, and demonstration of the correct BFT for the mothers during ANC, PNC, and for all mothers with sick young infant according to the Integrated Management of Neonate and Childhood Illness (IMNCI) guidelines [1, 16, 19–21]. Ethiopia has also adopted WHO IMNCI guidelines, which contain breastfeeding technique assessment components for sick young infants [21]. More recently, in 2019, Ethiopia included ineffective breastfeeding technique as a diagnostic criterion of severe acute malnutrition for infants less than 6 months in the national guideline for the management of acute malnutrition [22].

Despite these, the reduction of infant and preventable under-five mortality is still the main focus of several national and international health organizations. For instance, the Ethiopian government has targeted end childhood under nutrition by 2030, with a commitment to the ‘Seqota’ Declaration. One way to achieve the aforementioned aimed to promote effective breastfeeding during infancy and early childhood. This study can demonstrate the magnitude and factors contributing to effective BFT which helps to attain the national goal. Though there is a study conducted at’ Dire Dawa city, Ormiya region, Ethiopia‘, the study failed to consider the suckling technique and were not applied an observational data collection technique. Therefore, this study aimed towards assessing BFT and associated factors among lactating mothers in Gondar town, Northwest Ethiopia. By doing this, it would be helpful for community, health care providers as a body of knowledge, programmers, and policy makers in designing strategies for the enhancement of effective breastfeeding in both the study area and similar settings.

## Methods

### Study design and setting

An institution based cross-sectional study was conducted at public health facilities in the Gondar town from February 20 to March 20, 2020. Gondar town is founding in the central Gondar zone, Amhara regional state; north of Lake Tana and southwest of the Simien Mountains. It is far from a distance of 727 km from Addis Ababa, the capital city of Ethiopia. There are one comprehensive specialized hospital and eight health centers which gives services for more than 5000,000 people. According to the Gondar town health office, 652,940 patients visit all the nine governmental health facilities in the town per year, of whom 21,527 are children less than 5 years of age [23]. Each facility’s provided vaccination according to the EPI (Expanded Program for Immunization) schedule for children under 2 years with the monthly flow rates 68up to 632.

#### Participants of the study

All lactating mothers with their infant (mother-infant dyad) visiting the governmental health facilities of Gondar town were the source population. Lactating mothers who came to the EPI unit with their baby during the data collection period and who had their infants/children less than 15 months were included in the study. However, Mothers who were not able to breastfeed their baby due to any known medical problems, infants who were bottle feeders, and infants brought to the EPI unit by other attendants rather than their mother were excluded from the study.

#### Sample size and sampling procedure

The sample size was calculated by using single population proportion formula based on the following statistical assumptions; *P* = 43% (Proportion of effective BFT from the study conducted in Harar city, Harari regional state, Eastern Ethiopia 2017) [24], 95% confidence level, and 5% margin of error. Based on this formula and after adding a 10% non-response rate the total sample size was 414. Thus, data were collected from 414 mother-infant dyads attending the immunization unit.

A stratified simple random sampling method was employed to approach study participants from the eight public health centers and one comprehensive specialized hospital in the town. All nine health facilities were included in the study and the total sample size was allocated proportionally to each facility. To select the desired sample of mother-infant pairs among attendants of all health facilities, the average number of mothers who visited the EPI unit of each health facility for immunization within the last 3 months before the study was identified from the client registrations. On the bases of this, the expected client flow rate during the study period (1 month) was estimated to be 2512. Then, the sampling interval (k) was calculated by dividing the expected number of the mothers visiting the unit during the study period (N) for the determined sample size (n) of respondents (2512/414 = 6). Finally, using a systematic random sampling technique, one in every six mother-infant pairs was selected until the required sample was met.

#### Data collection tool and procedure

The required data were collected using WHO B-R-E-A-S-T- Feed Observation checklist and a face-to-face structured interviewer-administered questionnaire which is adapted and modified from the previous studies [17, 24]. The observational checklist was used to determine the correctness of positioning, attachment to breast, and suckling during breastfeeding. Other data concerning socio demographic profile, infant characteristics, and maternal characteristics were collected by interviewing the mother. The English version of the tool was translated to the local language (Amharic) and to see the consistency of the tool again back to the English. The pre-test was done on the twenty respondents at “Maksegnit” health center. Inter-observer variability was also checked and adjustment was made based on the results of the pre-test.

Eight diploma nurses were recruited as data collectors and two BSc nurses as supervisors from other health facilities than that included in the study. Orientation and training were given for all data collectors and supervisors for 1 day on how to collect data, and how to observe and record the positioning, attachment, and suckling using video, images, and demonstration. After obtaining informed verbal consent, concealed observation of BFT was made for 4 min [21] by asking the mother to put her baby to her breast. If the infant had been fed recently and refused to feed, then the mother was asked to wait when the infant would like to suckle again while filling socio demographic data.

After observation, data collectors manually recorded the mother and infant’s positioning, attachment to the breast, and suckling as per the WHO B-R-E-A-S-T Feed observation form. Each criterion under the three techniques; positioning (four criteria), attachment (four criteria) and suckling (three criteria) carry 1 point and the summation of criteria under each technique was recorded on the space provided to quantify the observation.

#### Operational definitions

##### Breastfeeding technique

The composite of positioning, attachment, and suckling while breastfeeding [24]:

***Positioning***: Physical alignment or the way a mother holds her baby.

***Attachment***: The way a baby takes the breast into his mouth and whether the infant has enough areola and breast tissue in the mouth.

***Suckling***: The action by which a baby removes milk from the breast.

##### Effective breastfeeding technique

The achievement of the combination of at least two criteria from positioning, three criteria from the attachment, and two criteria from suckling while mothers’ breastfeeds their infant [17, 24]. (Table 1).

**Table 1:** Criteria and grading system for positioning, attachment, and suckling among lactating mothers visiting health facilities in Gondar town, Amhara Regional State, Northwest Ethiopia, 2020

Criteria for correct body positioning		
Baby’s body close to the mother’s body		
Baby body and neck straight		
Baby facing toward the mother’s breast		
The whole body supported by the mother		
Criteria for grading body positioning:	**Grade**	**Score**
None or only one out of four criteria has been fulfilled	Poor	0–1
Any two of the four criteria has been fulfilled	Average	2
Three/all the four criteria for body positioning were fulfilled	Good	3–4
Criteria for the correctness of attachment		
More areola is seen above the baby’s top lip		
The Baby’s mouth is wide open		
Baby’s lower lip turned outwards		
Baby’s chin touching the breast		
Criteria for grading of correct attachment:	**Grade**	**Score**
None of or only one out of four criteria has been fulfilled	Poor	0–1
Any two of the four criteria has been fulfilled	Average	2
Any three or all the four criteria has been fulfilled	Good	3–4
Criteria for the correctness of effective suckling:		
Slow suckling		
Deep suckling		
Sometimes pausing		
Criteria for grading of effective suckling:	**Grade**	**Score**
None or only one of the three criteria has been achieved	Ineffective	0–1
Any two or all three criteria has been achieved	Effective	2–3

**Slow suckling**: suckling rhythm of about one suck per second [23].

**Deep suckling**: the baby’s cheeks shouldn’t draw inward and are rounded during a suckling, evidenced by visible or audible swallowing after every one or two sucks [23].

**Breast problems:** are problems such as ***Engorgement*** = painful and swollen breast and when the milk doesn’t flow well, ***Crackle*** = break in the skin, sometimes called a fissure, ***Inverted nipple*** = a nipple which goes inward instead of sticking out, ***Sore nipples*** = pain in the nipple and areola when the baby feeds.

**Bottle feeding**: Feeding an infant from a bottle whatever is in the bottle; in addition to breastfeeding (mixed feeding) [25].

**Gestational age**: Preterm < 37 weeks, term 37–42 weeks.

**Pacifier** - is a rubber plastic, or silicone nipple given to an infant to suck upon [24].

**Parity**: primipara- a mother who has given birth to only one time; multipara-a mother who has given birth 2 or more times.

#### Data processing and analysis

After checking for completeness, data were coded and entered into Epi-Data version 4.6 software and then exported to SPSS version 20.0 for further data analysis. Descriptive statistical analyses were computed and important variables were presented by percent, frequency, tables, and figures. The outcome variable breastfeeding technique (composite of positioning, attachment, and suckling) was dichotomized and coded as “1” for effective BFT and “0” for ineffective BFT. To call effective BFT the total score of the three composite variables should be greater than or equal to seven (≥7) and the BFT is ineffective if the score is less than seven (< 7). Standardized residuals were analyzed to check the presence of outliers, as well as multi-collinearity, was checked using the variance inflation factor, and variables with variance inflation factor greater than ten were removed. Model fitness was tested using the Hosmer-Lemeshow’s goodness of fit test.

To examine the association between the independent and outcome factors, initially, bi-variable logistic regression analysis was conducted. Independent variables with a *p*-value ≤ of 0.25 in the bi-variable logistic regression analysis were included in the multivariable logistic regression analysis to control for all possible confounders and identify the significant factors. The strength of the association between the dependent and independent variables was measured using adjusted odds ratios with 95% CI. And if OR < 1 which means mothers not performed effective breast feeding technique whereas OR > 1 mothers applied effective breast feeding technique. The p-value of 0.05 or less was used for the final interpretation of statistical significance.

## Results

### Socio-demographic characteristics of the respondents

Out of a total of 414 lactating mother-infant pairs were participated in this study, of which 410(99%) were responded. Near to 91 %, 372(90.7%) of the respondents were urban dwellers. In this study, the mean ages of the respondents were 27.3 + 4.8 SD years old. The majority of study participants 391(95.4%) were married and two-thirds 272(66.3%) of them were housewives. Regarding their level of education, more than one third 153(37.3%) of them had attended up to secondary school **(**Table 2**)**.

**Table 2:** Socio-demographic characteristics of lactating mothers attending EPI in public health facilities in Gondar town, Amhara Regional State, Northwest Ethiopia 2020 (*n* = 414)

Variables	Frequency	Percent
**Residence**		
Urban	372	90.7
Rural	38	9.3
**Age of mother in year**		
< 20	29	7.1
20–25	130	31.7
26–30	170	41.4
> 30	81	19.8
**Ethnicity**		
Amhara	352	85.9
Kimant	38	9.3
other^a^	20	4.9
**Religion**		
Orthodox	348	84.9
Muslim	56	13.7
Protestant	6	1.5
**Marital status**		
Married	391	95.4
Divorced	17	4.1
other^b^	2	0.5
**Occupation**		
housewife	272	66.3
government employee	73	17.8
self-employee	44	10.7
other^c^	21	5.1
**Educational status**		
has no formal education	54	13.2
grade 1–8	91	22.2
grade 9–12	153	37.3
diploma and above	112	27.3

^a^Oromo, Tigre, Wolayta, and Hadiya

^b^Single and widowed

^c^NGO employee, daily laborer, and student

### Maternal obstetric and infant characteristics

Two hundred thirty (56.1%) of the mothers had previous breastfeeding experience, and 231(56.3%) of them were multiparous. The majority of 388(95%) mothers had ANC follow-up visits during their last pregnancy. Significant number of mothers, 386(94%) gave birth to their current baby at health facilities, however, many of the mothers 322(78.5%) had not received counseling about BFT during their ANC follow-up. Abundant number of participants, 387(94.4%) had not breast related problems. Related to infant’s or children’s characteristics, 284(69.3%) of them were found within the age range 0–6 months. Small number of infants, 21(5.1%) were used bottle feeding, and 70(17.1%) were used pacifier **(**Table 3).

**Table 3:** Maternal obstetric and infant characteristics of participants in public health facilities in Gondar town, Amhara Regional State, Northwest Ethiopia 2020 (*n* = 414)

Variables		Frequency	Percent
**Previous breastfeeding experience**	Yes	230	56.1
	No	180	43.9
**Breastfeeding experience duration in a years**	< 2	94	22.9
	2–5	108	26.3
	> 5	28	6.8
**Parity**	Primipara	179	43.7
	Multipara	231	56.3
**ANC**^a^ **follow up**	Yes	388	94.6
	No	22	5.4
**Counseling about breastfeeding technique**	Yes	88	21.5
	No	322	78.5
**Place of delivery**	Hospital	282	68.8
	health center	98	23.9
	private clinic	6	1.5
	Home	24	5.9
**Mode of delivery**	SVD^b^	290	70.7
	Assisted delivery	42	10.2
	C/S^c^	78	19.0
**Immediate counseling about BFT**	Yes	187	45.6
	No	223	54.4
**PNC**^d^	Yes	175	42.7
	No	235	57.3
**PNC visit frequency**	1 time	117	28.5
	2 and above	58	14.1
**Breast problem**	Yes	23	5.6
	No	387	94.4
**Sign and symptoms of a breast problem**	Crackle nipple	3	0.7
	Sore nipple	11	2.7
	Engorgement	8	2.0
	Inverted nipple	1	.2
**Gestational age**	Preterm	24	5.9
	Term	386	94.1
**Sex of the infant**	Male	213	52.0
	Female	197	48.0
**Age of infant/child**	0–6 months	284	69.3
	7–12 months	74	18.0
	13–15 months	52	12.7
**The baby started complementary feeding**	Yes	126	30.7
	No	284	69.3
**Feeding type**	Mixed	7	1.7
	normal	119	29.0
**The baby uses a bottle**	Yes	70	17.1
	No	340	82.9
**The baby uses a pacifier**	Yes	21	5.1
	No	389	94.9

^a^antenatal care

^b^spontaneous vaginal delivery

^c^caesarian section d postnatal care

### Breastfeeding technique status

**Fig. 1:**
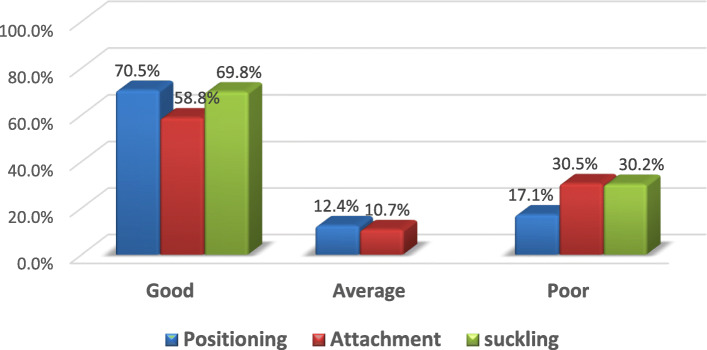
Breastfeeding technique status of lactating mothers visiting public health facilities in Gondar town, Amhara Regional State, Northwest Ethiopia 2020

The proportion of effective breastfeeding technique in the study area was [48% (95%; CI: 43.0–53.0%)]. The majority, 289(70.5%) of the mother exhibits good positioning. Infant attachment was good, and suckling was effective in 241(58.8%) and 286(69.8%), respectively (Fig. 1).

### Factors associated with breastfeeding technique

In both bivariate and multivariable logistic regression analyses, the age of the mother, parity, counseling about BFT during ANC follow-up, and immediately after delivery, as well as breast problem and bottle feeding, were significantly associated factors for BFT.

The odds of effective BFT was 70% less than (AOR = 0.3; 95% CI: 0.11, 0.83) among age group 18–20 compared to age group greater than 30 years. The probability of Effective BFT was 52% less than(AOR = 0.48; 95% CI: 0.26–0.88) among age group 26–30 years compared to age group greater than 30 years. The odds of effective BFT in primipara were 49% (AOR = 0.51; 95% CI: 0.32–0.82) lower compared to multiparous mothers. Similarly, the likelihood of having effective BFT was 45% less than [(AOR = 0.55, 95% CI: 0.32,0.95)] those mothers who have no counselling of BFT during ANC follow up, and 55% less than(AOR = 0.45; 95% CI: 0.29,0.70) mothers who had no immediate post-natal counseling compared to their counterparts. Moreover, the result of this study showed that effective BFT was 78% AOR = 0.22; 95% CI: 0.07–0.68) less in mothers with breast problem and 46% (AOR = 0.54; 95% CI: 0.30–0.97) less in bottle-feeders compared to their counterparts (Table 4).

**Table 4:** Bivariate and multivariable logistic regression analysis for breastfeeding techniques by maternal and infant factors among lactating mothers visiting Gondar town health facilities, Amhara regional state, northwest Ethiopia, 2020.(*n* = 414)

Variables		Effectiveness of BFT:		COR (95% CI)	AOR (95% CI)
		Effective N (%)	In-effective N (%)		
**Age of mother**	18–20	9(31.0)	20(69.0)	0.21(0.08–0.53)^*^	0.30(0.11–0.83)^**^
	21–25	53(40.8)	77(59.2)	0.32(0.18–0.58)*	0.45(0.23–0.87)**
	26–30	80(47.1)	90(52.9)	0.42(0.24–0.73)*	0.48(0.26–0.88)**
	> 30	55(67.9)	26(32.1)	1.0	1.0
**Marital status**	Married	193(49.4)	198(50.6)	1.0	1.0
	Other	4(21.1)	15(78.9)	0.27(0.09–0.84)^*^	0.34(0.10–1.13)
**Parity**	primipara	63(35.2)	116(64.8)	0.39(0.26–0.59)^*^	0.51(0.32–0.82)^**^
	multipara	134(58.0)	97(42.0)	1.0	1.0
**Counseling of BFT during ANC follow up**	Yes	58(65.9)	30(34.1)	1.0	1.0
	No	139(43.2)	183(56.8)	0.39(0.24–0.64)*	0.55(0.32–0.95)^**^
**Immediate PNC counseling**	Yes	115(61.5)	72(38.5)	1.0	1.0
	No	82(36.8)	141(63.2)	0.36(0.24,0.54)^**^	0.45(0.29,0.70)^**^
**Breast problem**	yes	4(17.4)	19(82.6)	0.21(0.07,0.63)*	0.22(0.07,0.68)^**^
	No	193(49.9)	194(50.1)	1.0	1.0
**Gestational age**	preterm	8(33.3)	16(66.7)	0.52(0.22,1.25)	0.46(0.18,1.20)
	term	189(49.0)	197(51.0)	1.0	1.0
**Bottle feeding**	yes	26(37.1)	44(62.9)	0.58(0.34,0.99)^*^	0.54(0.30,0.97)**
	No	171(50.3)	169(49.7)	**1.0**	**1.0**

*Bi-Variable significant (p-value < 0.05)

** multivariable significant (p-value < 0.05)

## Discussions

Ineffective breastfeeding technique is the leading cause of various problems related to breastfeeding and significantly affects both maternal and infants health [26, 27]. This study attempted to examine the magnitude and factors associated with BFT among lactating mothers who visited public health facilities in Gondar town. The finding of this study showed that just less than half [48% (95%, CI: 43.0–53.0%)] of mothers apply effective BFT, depicting the presence of poor breastfeeding technique. This is consistent with the reports of the studies conducted in Harar city, Ethiopia (43.4%), Libya (48%), Indonesia (46.7%), and western Denmark (50%) [17, 24, 28, 29].

The proportion is considerably higher than the findings of the study done in the south Ari district of Ethiopia (36.5%) [30]. This variation might be because of the difference in the composition of study participants. For instance, a fewer portion of younger mothers was participated in the current study, unlike Ari district study participants. The proportion is, however, lower than the studies conducted in Nigeria (71.3%) and West Bengal/Kolkata in India(74%) [31, 32]. This is probably due to the variation in the data collection method and target population. The data collection method was not direct observation in the Nigerian study, which may lead to the over-reporting of the practice. In the Bengal study, only mothers in the postnatal ward were included in the study.

Younger mothers aged 18–20 and 20–26 years old were 70 and 52% less likely to practice effective breastfeeding techniques respectively, compared to mothers whose ages were greater than 30 years. This indicates that young mothers are offering breastfeeding to their infants’ inappropriately, which could ultimately adversely affects their health. Similar findings were reported from studies done in a teaching hospital in coastal Karnataka, India, Indonesia, and Libya [10, 17, 33, 34]. This shows an increase in mothers’ age rises the experience of BFT and their confidence in child handling.

The present study shows that the odds of effective BFT decrease almost by half (49%) in primipara compared to multiparous mothers. This shows the use of BFT is poor in women giving birth for the first time. This is in line with the finding from studies in India, Australia, and Ethiopia [3, 30, 35, 36]. This could be due to a reason multiparous mothers might have frequent BFT counseling and exposure previously, which may enable them to acquire and master the skill of breastfeeding, unlike primipara.

This study also showed that counseling about BFT had a significant contribution to effective BFT. The odds of effective BFT were 45% less likely in mothers who had no counseling during antenatal care follow-up compared to those who had counseling. This finding remarks that ANC follow-up has added postpartum purposes beyond assessing pregnancy risks and giving appropriate care. Similar findings were reported from a study done in Karnataka India, Libya, Harar city, and Ari southern Ethiopia [17, 24, 30, 34]. Antenatal counseling may enhance the mother’s knowledge and help them to convert this knowledge to practice as early as possible after delivery, whether they get immediate post-natal counseling or not.

Similarly, the usages of effective BFT were 55 % less likely in mothers who had no immediate postnatal counseling compared to their counterparts. This suggests that the immediate postnatal period is the most critical time for BFT counseling since all the mother’s attention is toward her baby during this time. This findings consistent with study done in northern India, Saudi Heraa general hospital, and Harar [24, 37, 38].. This is due to the direct observation of breastfeeding by a health care provider as a routine activity and repeated demonstration of breastfeeding techniques.

Moreover, mothers who had breast problems like engorgement, mastitis, and crackle were 78% less likely to display effective BFT than those mothers without breast problems, which means the prevention of breast problems is crucial for successful breastfeeding. The same is true in the finding of the studies conducted in Libya, and Eastern and southern Ethiopia [17, 24, 30]. This could be related to the fear of pain and discomfort felt by the mother with the problem during breastfeeding. Similarly, in the present study, there was a significant association between BFT and bottle-feeding. Mothers who have bottle feeding practice were 46% less likely to demonstrate effective BFT., This is in accordance with the previous study conducted in Brazil, BFT was significantly affected by bottle-feeding [39]. This might be due to the mothers who bottle-fed their baby wouldn’t frequently breastfeed their baby.

### Limitation of the study

Since the main data collection method was the use of an observational checklist the study might be subjected to the Hawthorne effect, though, this effect was tried to be minimized by concealing the observation from the study participants. Lack of repeated observation of breastfeeding techniques may compromise the ascertainment of the breastfeeding technique status of the mothers.

## Conclusion

In conclusion, this study revealed that the proportion of effective breast feeding technique was slightly high compared to a study done in Harar, Ethiopia. The socio-demographic variable like the age of the mothers(older age) and other maternal characteristics including multi-parity, having counseling about breastfeeding technique during antenatal care follow-up, immediate postnatal counseling as well as not having breast problems and bottle feeding found to be an independent pronounced predictor of effective breastfeeding technique. Hence, special emphasis should be given to younger mothers and primipara mothers. Besides, preventing breast problems and working on increasing ANC and immediate postnatal counseling would improve BFT.

## Data Availability

All data and materials relevant to this study are available from the corresponding author whenever required.

## Abbreviations

BFT: Breastfeeding Technique
EPI: Expanded Program for Immunization
FMOH: Federal Ministry of Health
IMNCI: Integrated Management of Newborn and Childhood Illness
